# Subtle frequency matching reveals resonant phenomenon in the flight of Odonata

**DOI:** 10.1098/rsif.2024.0401

**Published:** 2024-10-23

**Authors:** C. Aracheloff, R. Garrouste, A. Nel, R. Godoy-Diana, B. Thiria

**Affiliations:** ^1^ Laboratoire de Physique et Mécanique des Milieux Hétérogènes (PMMH), CNRS UMR 7636, ESPCI Paris - PSL University, Sorbonne Université, Université Paris Cité, Paris 75005, France; ^2^ Institut de Systématique, Evolution, Biodiversité (ISYEB), CNRS UMR 7205, MNHN, Sorbonne Université, Paris 75005, France

**Keywords:** Odonata, insect flight, resonance, nonlinear mechanisms

## Abstract

In this work, we investigate the connection between the flight flapping frequency and the intrinsic wing properties in Odonata (dragonflies and damselflies). For such large flying insect species, it has been noted that the wingbeat frequency is significantly lower than the structural resonance of the wing itself. However, the structural resonance mechanism is often evoked in the literature for flying and swimming animals as a means to increase locomotion performance. Here, we show that the flight of Odonata is based on a nonlinear mechanism that strongly depends on the wingbeat amplitude. For large flapping amplitudes (as observed in natural flight), the resonant frequency of the wings decreases with respect to its value at low amplitudes to eventually match the wingbeat frequency used in flight. By means of this nonlinear resonance, Odonata keep a strong wing stiffness while benefiting from a passive energy-saving mechanism based on the dynamic softening of the wing.

## 1. Introduction

The flight capabilities of flapping flyers, especially insects, are very sophisticated [1–5]. Certain species, such as those of the Odonata, an order that includes dragonflies and damselflies, are capable of very complex flight dynamics: forward and backward flight, hovering and gliding, all with strong acceleration [6–8], controlled by four independent wings [9]. Despite the complexity of their flight behaviour, they rely on a relatively simple wing actuation mechanism based on direct flight muscles at the wing root that drives the flapping motion. Because there is no muscle on the wings, their structural deformation is completely passive [10,11].

The flight muscles are, therefore, heavily taxed, and flapping flight is associated with high energy expenditure. To reduce energy costs and ease the strain on the flight muscles, resonance mechanisms have often been proposed in the literature [1,12]. Two different resonance-based mechanisms are often evoked for flapping flyers. The first one, shared by bees and flies [13,14], consists of triggering a resonance in the thorax structure [2] that synchronizes the amplitude and the frequency of the beating wings with the motion of the thorax. The second one consists of taking advantage of the resonance of the wing structure itself [12,15–17]. If the frequency of the flight muscle that drives the flapping movement matches the natural frequency of the wing structure, the flyer can use this resonance to significantly increase the amplitude of the beating without increasing energy expenditure. The basic idea of this mechanism is appealing because of its simplicity.

In animals such as Odonata, the amplitudes of the beating motion are very large, i.e. of the order of the wing size itself. It is well known that vibrating systems operating at high amplitudes experience strong nonlinear effects with direct consequences on the frequency response [18–20], but such studies have not been carried out on real insect wings. However, especially in large insect species, a significant discrepancy is observed between the beating frequency measured during normal flight in nature and the natural frequency of the wing structure. The flapping frequency is always well below the wing resonant frequency [21–24]. The idea of resonance-based mechanisms for flight may be challenged by this observation. However, the characterization of the wing natural frequencies in insects is often obtained using quasi-static bending test measurements [24–26] or small-amplitude vibration set-ups [17,21,27].

In this article, we address the problem of the vibrational behaviour of real Odonata wings. First, we measure the structural natural frequencies of the wings of several dragonfly and damselfly species using small-amplitude vibration tests (i.e. in the linear regime) and compare them with the flapping frequencies found in the literature. Then, we focus on two species of Pseudostigmatidae, a family of Zygoptera, to perform an in-depth vibration study at large amplitudes. We demonstrate that the wing as a structure exhibits a strong *softening* nonlinear effect that shifts the effective natural frequency to lower values as the amplitude increases. More remarkably, we show that for flapping amplitudes comparable to those observed during natural cruising flight, the nonlinear resonant frequency of the wing matches the range of the flapping frequencies used by the insects, enabling the existence of a resonance-based regime. We discuss these unprecedented findings not only in the context of understanding Odonata flight but also concerning the strong implications for bio-inspired artificial flapping flyers [28–34].

## 2. Results

**Figure 1.**
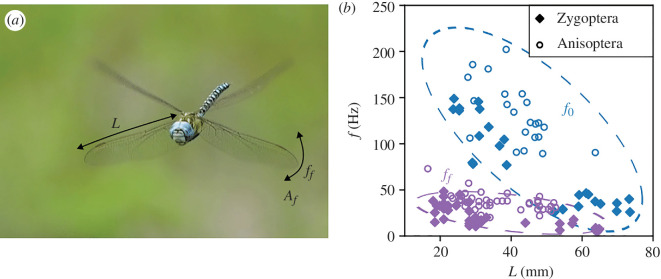
(*a*) Picture of *Aeshna mixta* (Odonata: Aeshnidae) showing the wingspan L and the kinematic parameters during flight $A_{f}$ and $f_{f}$, respectively, the amplitude of the wing tip displacement and the flapping frequency. (*b*) Comparison of the observed values of flapping frequency during flight $f_{f}$ (purple symbols) found in the literature (see table 1) and the wing structural natural frequency of oscillation $f_{0}$ (blue symbols) measured experimentally in the linear regime for several Odonata species, as a function of the wingspan.

A large dataset of wingbeat frequencies in flight $f_{f}$ for several Odonata species was compiled from the literature (see Material and methods, table 1). The frequencies $f_{f}$ are plotted in figure 1*b* as a function of the wingspan L and compared to natural structural frequencies $f_{0}$ obtained experimentally using small-amplitude vibration tests on 52 wings of 23 dry specimens from seven Odonata families. In these tests, the wings with their bases attached are removed from the body and glued to a wing clamping system, ensuring a stable support at the root of the structure. For each test, a wing is mounted on a precision shaker, allowing a fine tuning of the frequency and amplitude of the vibrations (see §4). The motion imposed at the wing root is a sinusoidal: $d(t)=A_{i}\cos(\omega_{i}t)$, where $A_{i}$ is the forcing amplitude and $\omega_{i}=2\pi f_{i}$ with $f_{i}$ the the imposed frequency. Figure 1*b* shows that for an equivalent wingspan, the structural natural frequency is higher than the flapping frequency, as described in the literature [21,22,24]. A decrease of both the natural frequency $f_{0}$ and the flight frequency $f_{f}$ is observed for increasing values of the wingspan L.

**Table 1.** Observed flight frequency in literature data about Odonata flight [17,24,35–44], where $f_{f}$ is the reported flapping frequency. Depending on the paper, we have the standard deviation (±), the range of observed frequency (minimum–maximum value), the wingspan (*L*) with the same nomenclature as for $f_{f}$ and the stroke angle (2ϕ) previously defined in the article. All data in the same row come from the article cited in the flight frequency ($f_{f}$) column if there are no additional references. We use the flight data of *Megaloprepus caerulatus* (Odonata: Pseudostigmatidae) marked ♦ to calculate the wing tip displacement shown in figure 2 using A/L=sin⁡(ϕ).

suborder	family	species	$f_{f}$ (Hz)	*L* (mm)	stroke angle (2ϕ)
Anisoptera	Aeshnidae	*Aeshna cyanea*	42.9±5.3 [40]	48.0	
			32.4±5.2 [35]	44–52	
			22±3.2 [35]	44–52	
			36 [36]	45.1±1.1	
			35 [36]	45.1±1.1	73/84
			35 [36]	45.1±1.1	90
			29–46.4 [37]	13–60	90
			29–46.4 [37]	25–50	90
		*Anax imperator*	29.2–47.5 [36]	47.5	
			36.5 [36]	47.5	
			29 [36]	47.5	73/86
		*Anax junius*	36 [36]	51–53	90
		*Anax parthenope julius*	28.3–35.8 [38]	51.2±1.82	
			27 [24]		
		*Anax junius*	29 [39]	50.5 [45]	
	Corduliidae	*Cordulia aenea*	47.6±5.5 [35]	29–34	
			22.5±2	29–34	
		*Epitheca cynosura*	43 [39]		
	Libellulidae	*Libellula quadrimaculata*	33.2±6.2 [35]	32–36	
			20.1±2.1 [35]	32–36	
			36.4±8.6 [40]		
		*Leucorrhinia rubicunda*	41 [36]	28.9±0.98	90 (hovering)/150 (take off)
		*Micrathyria atra*	39 [39]	33.0 [46]	
		*Nesciothemis farinosa*	57.3±9.3 [40]		
		*Neurothemis fluctuans*	40.8±15.4 [40]		
		*Neurothemis ramburii*	33.1 [41]	33±0.3	56.8/71
		*Orthetrum cancellatum*	38–59.5 [36]	38.9±1.5	80–90 (hovering)/130 (take off)
			46.4 [36]	38.9±1.5	
		*Pachydiplax longipennis*	41.7–23.8 [42]	30.9±2.3	
			41.7–33.3 [42] (yaw turn)	30.9±2.3	95–100
			33.3–23.8 [42] (pursuit)	29.8±1.9	
		*Pantala flavescens*	18.85–38.01 [38]	42.71±2.3	
		*Perithemis tenera*	73 [36]	16.5	
		*Sympetrum flaveolum*	36.63±1.85 [17]	27.5 [51]	
		*Sympetrum sanguineum*	38.7±0.82 [43]		90.5±4.95 (64.1–107)
			39.2±1.61 [43]		101.56±3.92 (88.5–115.8)
		*Sympetrum danae*	43.5 [36]	23	
		*Sympetrum vulgatum*	32.3 [36]	27	
		*Sympetrum baccha matutinum*	31 [24]		
		*Tramea lacerata*		49	
	Libellulidae	*Trithemis arteriosa*	46.3±17.6 [40]	27	
	Macromiidae	*Macromia taeniolata*	31 [39]	52.5 [47]	
Anisozygoptera	Epiophlebiidae	*Epiophlebia superstes*	40–52 [37]	30–33	67–97
Zygoptera	Calopterygidae	*Calopteryx atrata*	15 [24]		
		*Calopteryx splendens*	17.3±6.6 [40]	31.5 [48]	
			19.9±1.21 [43] (forewings)	29.9±0.5	120.1 7.51
			20.3±1.26 [43] (hindwings)	29.9±0.5	121 6.95
			18.6±5.7 [35]	27–32	
			15.4±2.1 [35]	27–32	
			16–38 [36]	28.2±0.8	
			14.1–19.2 [36]	28.2±0.8	125
			13.5–17.8 [36]	28.2±0.8	130
			37 [36]	28.2±0.8	68.5
			11.1–19.2 [36]	28.2±0.8	110
		*Calopteryx virgo*	10.7–16 [36]	30.0 [48]	110
		*Calopteryx virgo and splendens*	10.7–19.2 [37]	30–54	110
			10.7–19.2 [37]	30–36	110
	Coenagrionidae	*Coenagrion puella*	38±5.2 [40]	18 [48]	
			25.5±2.6 [35]	15–22	
			18.2±1.6 [35]	18–24	
		*Cercion calamorum calamorum*	15 [24]		
	Calopterygidae	*Neurobasis chinensis*	20.2±6.8 [40]	32–34 [49]	
		*Matrona cyanoptera*	14.3 [41]	44±0.5	68.4/85.4
Zygoptera	Platycnemididae	*Platycnemis pennipes*	32.8 [36]	19.8	
	Lestidae	*Lestes viridis*	45±3.7 [40]		
			28–37.5 [36]	22.2	
			32.6 [36]	22.2	118
			32.8 [36]		117
			33 [37]	9–29	117
			33 [37]	22–27	117
	Chlorocyphidae	*Chlorocypha cancellata*	35.3±1.1 [44] (straight forward flight)		120
			48.3±5.7 [44] (threat display)		120
			43.1±4.1 [44] (chased from its perch)		
			29.5±3 [44] (threat display)		95/150
			42.9±3.1 [44] (courtship display)		85
	Pseudostigmatidae	*Mecistogaster ornata*	6.3–22.5 [36]	53.7±3.7	
			13.5 [36]	53.7±3.7	
			13.5 [36]	57.2±2.3	
		*Mecistogaster linearis*	17.8 [36]	57.6±1.7	
		*Megaloprepus caerulatus*	11.2±4.2 [40]		
			4.7–12.6 [36]		
			7.2 [36]	64.8±9.3	
			8.8 [36]	63.8±3.1	
			7.7 [36] ♦	64.8±9.3	133
Zygoptera	Pseudostigmatidae	*Megaloprepus caerulatus*	7.7 [36] ♦		103
			6.9 [36] ♦		128
			6.4 [36] ♦		132.4
			6.1 [36] ♦		106
			5.9 [36] ♦	63.8±3.1	121

**Figure 2.**
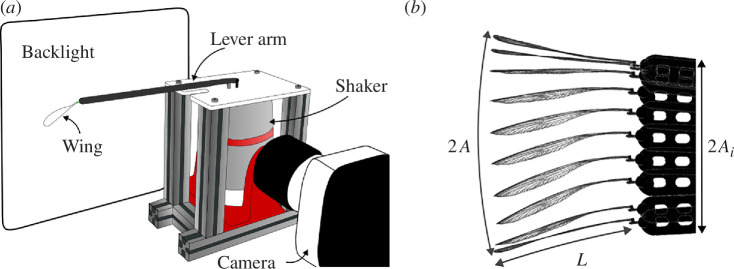
(*a*) Sketch of the experimental set-up for the study of large-amplitude displacement. The shaker allows to impose a displacement at the root of the lever arms, and the wing is glued to the attachment piece and clamped at the end of the lever arms. The high-speed camera records the wing during the experiment in front of a backlight, which allows a good contrast. (*b*) An example of images obtained using the set-up described in (*a*) for a hindwing of *Microstigma rotundatum* (Odonata: Pseudostigmatidae), with *A* the amplitude displacement of the wing tip, $A_{i}$ the forcing amplitude imposed at the wing root and L the wingspan.


Four specimens of the family Pseudostigmatidae for which the wing resonance frequency is below 50 Hz were chosen to perform a second vibrational study with large-amplitude forcing (see figure 2 and §4 for details) in order to obtain wing tip displacements similar to those observed in flight. A broad range of imposed displacements was studied, from very small (approx. 0.25 mm) to very large amplitudes (approx. 4 cm) of the order of magnitude of the wingspan. We define the reduced amplitude as

(2.1) $$\begin{array}{ccc} & A^{*}=\frac{A-A_{i}}{L}\;, & \end{array}$$

where A is the displacement of the wing tip and $A_{i}$ is the forcing displacement at the root of the wings (see figure 2*b*), and the reduced frequency as


(2.2) $$\begin{array}{ccc} & f^{*}=\frac{f_{i}}{f_{0}}\;. & \end{array}$$

**Figure 3.**
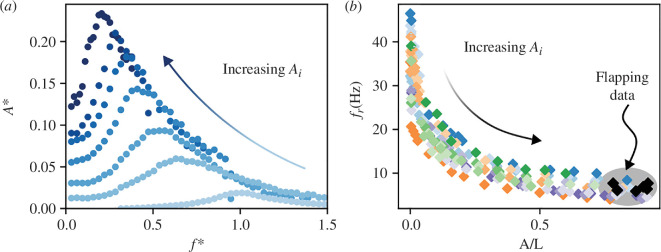
(*a*) Rescaled amplitude $A^{*}$ of the wing tip for a hindwing of *M. rotundatum* (Odonata: Pseudostigmatidae) as a function of the imposed reduced frequency $f^{*}$. The natural frequency in the linear regime is $f_{0}=32$ Hz in this case. The arrow shows the shift of the resonant frequency $f_{r}$ toward lower frequencies for increasing forcing amplitudes $A_{i}$. (*b*) Compilation of the resonant frequencies $f_{r}$ obtained experimentally for 14 wings from four specimens of two species of Pseudostigmatidae as a function of the normalized wing tip displacement. Each colour corresponds to a different wing. The arrow indicates that the resonant frequency decreases when the amplitude of displacement increases until it tends to the flapping frequency of Pseudostigmatidae from the literature [36] (for ♦, the normalized wing tip displacement was obtained using the stroke angle; see table 1).

Figure 3*a* shows typical responses of a wing of *M. rotundatum* (Odonata: Pseudostigmatidae). The peak of the lowest amplitude curve (light-shaded blue points) is directly related to the structural stiffness of the wing [50] and is used to define the linear resonance frequency $f_{0}$, which is ≈32 Hz in the case presented in figure 3*a*. This value is far from the flapping frequencies observed in this family of Zygoptera, which ranges from 4.7 to 22.5 Hz [36]. As the forcing amplitude $A_{i}$ increases, the resonant peak $f_{r}$ moves toward lower frequencies. This is a characteristic phenomenon of nonlinear vibrating structures [18]. For such systems, intrinsic nonlinearities deform the frequency response curve and shift the resonant frequency to either higher or lower values than the natural linear response. In the present case, the wing of our *M. rotundatum* experiences a softening behaviour at high forcing amplitudes: the wing responds as if its structural stiffness was lower than it is at low amplitude. It is worth noting that for large forcing amplitude $A_{i}$, the shift of the resonant peak toward lower values is significant. For this case, the resonant frequency $f_{r}\approx 7$ Hz is more than four times smaller than the natural frequency in the linear regime $f_{0}\approx 32$ Hz. We performed systematically the same experiment on two different species of Odonata from the Pseudostigmatidae family, *M. rotundatum* (Odonata: Pseudostigmatidae) and *Mecistogaster lucretia* (Odonata: Pseudostigmatidae), for which we tested four different specimens (three and one, respectively), corresponding to 14 different wings.

All the combined data are presented in figure 3*b*, which shows the resonant frequency of the wings as a function of the scaled amplitude A/L. The different colours correspond to the different wings tested. We have also added the flapping frequencies (and corresponding flapping amplitudes, black symbols) observed in nature during cruising flight and found in the literature for *Megaloprepus coerulatus* (Odonata: Pseudostigmatidae), a species in the same family as the one studied here, with a similar behaviour [36,51] (see §4 for details).

## 3. Discussion

As can be seen in figure 3*b*, all the resonance frequency data collapse into a single master curve. More importantly, as A increases, we see that the resonant frequency shifts continuously toward lower frequencies, eventually matching the actual wingbeat frequency observed in flight for Pseudostigmatidae. This is a remarkable result with important implications for understanding the flight mechanics of such large-scale flying species. Despite the high stiffness of their wings, which gives them great structural rigidity, these Odonata effectively use a nonlinear resonance mechanism to facilitate their flapping-powered flight. The nonlinear softening of vibrating systems is a result with implications not only for the understanding of the mechanisms at play in the locomotion of large flying insects but also for the design of future insect-inspired robots. Robotic wing designs can draw significantly from principles and mechanisms observed in biological systems [34], and the dynamic softening effect demonstrated here with large-amplitude flapping Odonata wings brings a real example of reconciling wing structural stiffness and energy optimization. Flapping in a resonance regime achieved by a nonlinear softening certainly means that the expensive high-amplitude wing flapping can be performed with less energy expenditure, as has been recently discussed in theoretical models of flapping wing drones with nonlinear springs [52].

This work has been carried out specifically on modern Odonata and should be extended to other large-scale species sharing the same large-amplitude flapping mechanisms, such as all the other dragonfly species, but also mayflies [13]. The detailed modelling of the nonlinear system based on the flight muscle actuation should be an interesting future development because the observed softening behaviour can stem from a combination of several factors that remain to be identified. The usual suspects are, on the one hand, the geometrical nonlinearities arising from the nonlinear strain–displacement relationship and the physical or material nonlinearities stemming from the nonlinear stress–strain behaviour of the wing material [53] and, on the other hand, an aeroelasticity problem, where nonlinear fluid–structure interactions can introduce softening effects due to added mass or damping effects. To differentiate structural effects from fluid interactions, experiments must be performed under both ambient and partial vacuum conditions. A preliminary test to consider this is shown in appendix B. The results reveal significant fluid contributions to the softening effect observed in this study, but a structural effect is not excluded. A full study of how to differentiate the aerodynamic and structural effects is, however, out of the scope of this paper, which focuses essentially on natural flying conditions in a normal atmosphere.

## 4. Material and methods

### 4.1. Details on the experimental apparatus

To perform the vibrational studies we used two set-ups.

#### 4.1.1. Small-amplitude tests

For the small-amplitude tests used to compute the structural frequencies $f_{0}$, the wings were attached to the axis of an LDS V201 Bruel & Kjaer™ shaker. The shaker was powered by an LDS PA25 amplifier of the same brand and driven by a sinusoidal signal with frequencies between 10 and 300 Hz. The wing is attached to the shaker using a small three-dimensional-printed clamp and the resulting amplitude of displacement at the root of the wing was ≈0.2mm.

#### 4.1.2. Large-amplitude tests

In order to study large displacements, we used a larger shaker (LDS V406) connected to a power amplifier (LDS PA100E). Using the wing clamp attached to the shaker axis, the obtained displacements were between 0.25 and 2 mm. The respective maximum frequencies were 50 and 45 Hz. Increasing amplitudes determine lower attainable frequencies. To be able to have an amplitude of several centimetres, we use a lever arm of 35 cm printed in Polymaker PolyMide™ (PA6CF), resulting in an amplitude of displacement between 2.5 and 40 mm, with maximum frequencies of 30 and 12 Hz, respectively.

Both shakers were controlled using a National Instruments card (NI 9263), the sinusoidal signal being generated by a custom Python code.

#### 4.1.3. Wing kinematics measurement

The wing displacement is measured using a laser sensor (optoNCDT 1402) with a range between 45 and 95 mm and a sampling frequency of 1.5 kHz. A small point was painted on the wing to have a good reflection for the laser measurement, since the wing membrane is transparent. The displacement data were collected using an NI cart 9205. For larger displacements, a high-speed Phantom Miro M120 camera was used, recording at 700 fps and also controlled with a Python code. To get a good contrast, a backlight panel was placed behind the wing. The acquired images were post-processed to track the displacement of the wing.

### 4.2. Field flight data

All the data from field observations have been taken from the literature [17,24,35–44]. A summary of the collected frequencies and stroke amplitudes is presented in table 1.

## Figures and Tables

**Figure 4.**
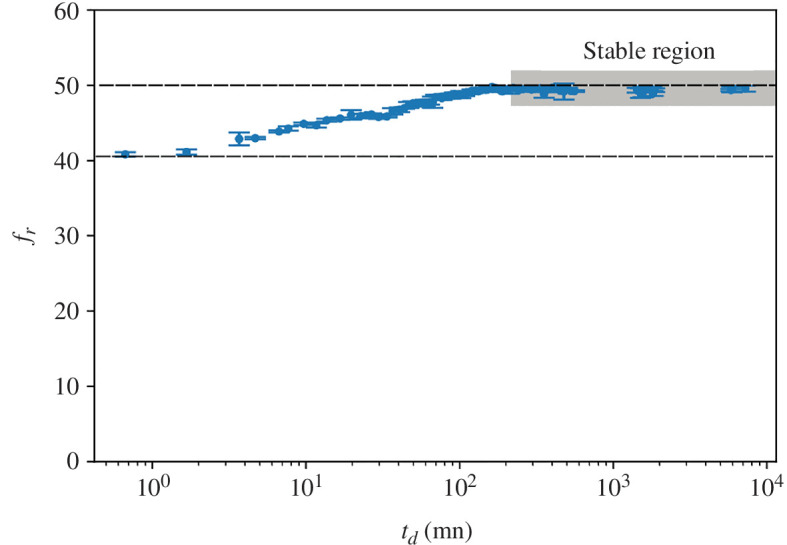
Resonant frequency $f_{r}$ as a function of time spent in ambient conditions $t_{d}$ obtained for a wing of *M. rotundatum* (Odonata: Pseudostigmatidae). The variation of $f_{r}$ between a humidified and dry wing is approximately 20%. This shift occurs in a period of 2 h. During this time, the mechanical property of the wing is not stable except if the experimental time is negligible in front of 2 h. It is not the case for our experiment; the acquisition time required to study the large amplitude (approx. 20–50 min amplitude^−1^). For this reason, we choose to work on dry wings, which are stable in ambient conditions, in order to have a stable object.

**Figure 5.**
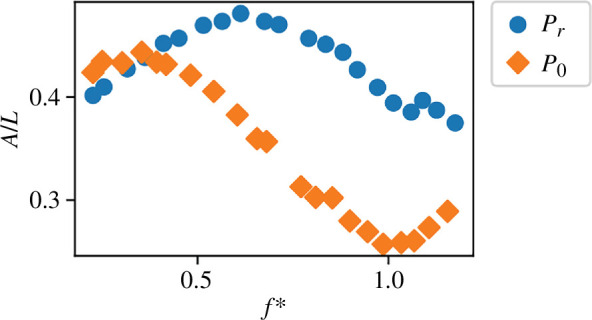
Normalized wing tip displacement (A/L) as a function of the imposed reduced frequency $f^{*}$ for a hindwing of *M. rotundatum* (Odonata: Pseudostigmatidae) in ambient (filled diamond) and partial vacuum condition (filled circle). The natural frequency in the linear regime is $f_{0}=21.4$ Hz in this case. A reduction of approximately 65% of pressure leads to an increase of $f_{r}$ of around 57% for A/L≈0.4.

## Data Availability

All data are available in the paper.

## Appendix A. Dehydration time study

Living wings are irrigated by the haemolymph. It was put in evidence the fact that wet and dried wings have different mechanical properties [54]. Even freshly collected wings taken from freshly dead specimens quickly dry. To get closer to the freshly collected wings, we tried to rehydrate our wings. However, as our experiments will be carried out under ambient temperature and humidity conditions, we need to control the dehydration time of our wings. The acquisition time required to study the large amplitude is around 20–50 min amplitude^−1^. We need to control the dehydration time of our wings to determine if we can work on rehydrated wings or if their mechanical properties change during the acquisition time.

We characterize a wing of *M. rotundatum* (Odonata: Pseudostigmatidae) placed in a humid environment (a hermetic box with water at the bottom) for more than 3 days using a damping method, which allows us to perform measurements in less than 1 min. We obtain the result shown in figure 4, which clearly shows a shift in the resonance frequency as a function of the time spent in ambient air. The dehydration time is estimated around 20 min. For this reason, we have chosen to work on dry wings, which are stable objects.

In addition, the variation of the resonant frequency between a humidified wing and the dry one is approximately 20%. This is less than nonlinear shift observed during our experiments changing the flapping amplitude, which can reach up to 50%.

## Appendix B. Fluid contribution study

The softening behaviour observed can have several origins as the inertia-elastic contributions and fluid contribution. An estimation of the role of each contribution can be obtained by performing a dimensional analysis on a simple model [55]. In our case, the ratio between inertia and fluid moments suggests that the fluid contribution is more significant than the inertia contribution due to the low surface density of Pseudostigmatidae wings (∼7 g m^−2^).

To assess the fluid contribution, we carried out large-amplitude experiments under both ambient conditions ($p_{0}=1$ bar) and partial vacuum ($p_{r}=0.325$ bar). The experiment is conducted in a hermetic box measuring 30 cm in length, constructed from 15 mm thick Plexiglas sheets [56]. A partial vacuum of $p_{r}=0.325$ bar is achieved using a LABOPORT N 820 pump. The vibrational set-up is a flapping set-up with a fixed angle $\alpha ∼38^{\circ }$ with imposed frequencies $f_{i}$ ranging from 5 to 25 Hz [57,58]. The motor is controlled via an L298N Arduino motor driver and by a National Instrument cart 9263 controller, using a Python code. Wing kinematics are measured using a high-speed camera (see §4).

We conducted vibrational tests for a hindwing of *M. rotundatum* (Odonata: Pseudostigmatidae) under both ambient and partial vacuum conditions (figure 5). The resonant frequency $f_{r}$ is around 7.5 Hz in ambient air and 13 Hz in partial vacuum. This result highlights the fluid contribution, which leads to an increased softening effect observed during our investigations without excluding a contribution from the structure to nonlinear behaviour.
